# Another Use for a Proven Drug: Experimental Evidence for the Potential of Artemisinin and Its Derivatives to Treat Alzheimer’s Disease

**DOI:** 10.3390/ijms25084165

**Published:** 2024-04-09

**Authors:** Eva Kiss, Stefan Kins, Karin Gorgas, Kinga Hajnal Venczel Szakács, Joachim Kirsch, Jochen Kuhse

**Affiliations:** 1 Institute of Anatomy and Cell Biology, University of Heidelberg, 69120 Heidelberg, Germany; gorgas@ana.uni-heidelberg.de (K.G.); joachim.kirsch@urz.uni-heidelberg.de (J.K.); 2 Department of Cellular and Molecular Biology, George Emil Palade University of Medicine, Pharmacy, Science and Technology of Târgu Mures, 540142 Târgu Mures, Romania; kingahajnalszakacs@gmail.com; 3 Department of Human Biology and Human Genetics, University of Kaiserslautern, 69120 Kaiserslautern, Germany; s.kins@biologie.uni-kl.de

**Keywords:** Alzheimer’s disease, artemisinins, multitargeting neurotherapeutics

## Abstract

Plant-derived multitarget compounds may represent a promising therapeutic strategy for multifactorial diseases, such as Alzheimer’s disease (AD). Artemisinin and its derivatives were indicated to beneficially modulate various aspects of AD pathology in different AD animal models through the regulation of a wide range of different cellular processes, such as energy homeostasis, apoptosis, proliferation and inflammatory pathways. In this review, we aimed to provide an up-to-date overview of the experimental evidence documenting the neuroprotective activities of artemi-sinins to underscore the potential of these already-approved drugs for treating AD also in humans and propose their consideration for carefully designed clinical trials. In particular, the benefits to the main pathological hallmarks and events in the pathological cascade throughout AD development in different animal models of AD are summarized. Moreover, dose- and context-dependent effects of artemisinins are noted.

## 1. Introduction

Alzheimer’s disease (AD) is the most common form of dementia characterized by progressive cognitive decline, pathological hallmarks of extracellular amyloid plaques formed by amyloid β peptides (Aβ), and intracellular deposits of hyperphosphorylated tau protein as neurofibrillary tangles in the brain [1]. However, a much better pathological correlate of cognitive dysfunction observed in AD is synaptic loss [2,3,4].

Sporadic forms of AD (i.e., late-onset) mostly start after age 65, whereas symptoms of the inherited forms of the disease usually develop before age 50. These “early-onset” forms compose less than 5% of all AD cases and are associated with mutations in genes involved in the amyloid-processing pathway, such as presenilin 1 (PSEN1) and 2 (PSEN2) and amyloid precursor protein (APP) [5]. The impaired APP cleavage caused by the mutations results in the increased production of amyloid beta (Aβ)_1–42_ peptides and failure in the Aβ clearance, thus providing an explanation for the Aβ accumulation in the brain of early-onset AD patients [1]. The identification of these gene mutations initiated the proposal of the “amyloid cascade hypothesis” in the early 1990s. It suggests that the generation and aggregation of Aβ are the initiator and, further, central factors in the cascade of cellular and molecular events that, ultimately, lead to AD [6,7]. Further supporting this theory, both genetic (e.g., ApoE4, TREM2, RIN3, CLU and PTK2B) and nongenetic (e.g., diabetes and obesity) risk factors for late-onset AD have also been identified to influence Aβ generation and/or Aβ clearance [8].

Aβ is released from APP through the amyloidogenic pathway after sequential clea-vage by β-secretase (BACE1) and γ-secretase during the course of its trafficking to the cell membrane. In addition to Aβ, other molecules such as sAPPβ (a soluble ectodomain of APP), CTFβ (C-terminal fragment β or C99) and AICD (APP intracellular domain) are also formed in the amyloidogenic pathway. Along the nonamyloidogenic pathway, membrane-anchored APP is cleaved by α-secretase within the Aβ sequence, thus preventing Aβ production [1,9]. Under physiological conditions, the nonamyloidogenic pathway predominates, and augmentation of the amyloidogenic pathway results in increased Aβ generation and AD pathogenesis. Under pathological conditions, there is also a change in the Aβ_1–42_/Aβ_1–40_ ratio in favor of an increased level of Aβ_1–42_, exhibiting decreased solubility and increased tendencies for fibril assembly and plaque formation [1,9]. After secretion, these Aβ peptides spontaneously aggregate and deposit into oligomers, fibrils, and plaques, which then progressively induce a series of events, including synaptic failure, aberrant proteostasis, such as tau phosphorylation and fibrillary tangle formation, cytoskeletal abnormalities, impaired energy homeostasis, DNA and RNA damage, increased inflammation and neuronal death [1]. All of these processes can also represent a link to the alteration of different forms of synaptic plasticity, changes that apparently develop long before the onset of clinical symptoms of AD [10]. Evidence supports that complex compensation mechanisms may work in the brain to maintain an almost normal cognitive performance for decades, with AD symptoms only starting when pathological cellular reactions are initiated [11]. Accordingly, the initial effects of “proteopathic” stress exerted by Aβ on various cells in the brain are physiological, involving primarily the lysosomal/endolysosomal system and, in particular, autophagy, to maintain homeostasis in the proteostatic network and various synaptic plasticity mechanisms. Even inflammatory reactions contribute initially to sustain homeostasis [11]. It is when these compensatory cellular homeostatic mechanisms fail that the clinical phase of the disease is initiated, and then it is no longer the accumulation of Aβ but other downstream events, such as neuroinflammation and tau accumulation, that are the main drivers of the neurodegeneration, eventually, in a synergistic manner. 

In addition to Aβ, other proteolytic fragments of APP may also participate in the pathogenesis of AD via differential mechanisms. Specifically, carboxy-terminal fragments (CTFs) of APP were found to exhibit even higher levels of neurotoxicity than Aβ [12]. CTFs were reported to translocate into the nucleus, where binding with Fe65 and CP2 affected the transcription of genes, including GSK-3 (glycogen synthase kinase-3), resulting in increased tau phosphorylation and the formation of neurofibrillary tangles [13]. More recently, overproduction of CTFs was indicated to cause alterations in mitochondrial structure and function, as well as failure of basal mitophagy, in vivo [14]. 

Another interesting aspect of AD pathogenesis is the demonstration that APP and APP cleavage products have important physiological roles, raising the question of whether their loss contributes to AD development [15,16]. The extracellular proteolytic protein of APP (sAPPα) [17,18], as well as Aβ, exert neuroprotective effects and have neurotrophic roles in synaptogenesis and neurogenesis [19,20]. For example, Aβ monomers can induce pathways mediated by the CREB (cAMP-response element-binding protein)-induced transcription of BDNF (brain-derived neurotrophic factor), known to be involved in adult hippocampal neurogenesis [21]. Further, it has been suggested that a hormetic mechanism for Aβ may operate, thus exerting opposing effects at low and high concentrations. Low concentrations of Aβ strengthen both synaptic plasticity and memory, whereas high concentrations result in the well-known impairment of cognition [19]. Neurotoxic Aβ aggregates are connected with damage to the blood–brain barrier (BBB), since low levels of Aβ peptides may function as a seal that maintains the BBB’s integrity [22]. Moreover, CTFs seem to be essential for endosomal and lysosomal functions and have a regulatory role in autophagy and mitochondrial function [14], whereas full lengths APP as a cell surface protein fulfill important functions for synaptic homeostasis and participate in signal transduction [17,23]. 

Thus, considering the complexity of AD and the failure or only very limited clinical effects of most single-target therapies [24,25], it seems essential that future therapeutic approaches target several pathogenic factors of the disease and, so far as possible, in a stage-dependent manner [8,26]. This includes exploring the potentials of natural products with neuroprotective effects that have a multitarget capacity and mild adverse events. Recent research has explored the potential benefits of specific floral extracts and by-products in the context of AD. For instance, some studies have evaluated the roles of flowers, such as rose x hybrida petals [27] and tubalghia volacea petals [28]. Certain by-products, including olive leaves, have also shown promise in combating AD [29]. Moreover, the potential therapeutic effects of honey in the context of AD have been explored (e.g., [30,31]). Additionally, over the several last years, an increasing number of studies in animal models have indicated the capability of different plant extracts used in traditional Chinese medicine to treat AD [32,33,34,35,36]. Moreover, an oral compound extracted from brown seaweed was approved in China, in 2019, to treat patients with mild to moderate forms of the disease [37]. Artemisinins represent another promising member of these plant-derived groups of drugs, holding the additional privilege of already being approved for the treatment of malaria with a known clinical safety and efficacy. 

## 2. Artemisinins: Chemical Structure, Pharmacokinetic Behaviors and Antimalaria Activities

Artemisinin and its derivatives (collectively termed as artemisinins) are sesquiterpene lactones derived from the plant sweet wormwood (Artemisia annua), which has been applied in traditional Chinese medicine to treat “fever”. Currently, artemisinins are first-line drugs in the treatment of malaria caused by Plasmodium parasites [38]. For the isolation and discovery of artemisinin, the active compound of the plant, as an antimalarial drug, the Chinese scientist Tu Youyou received the Nobel Prize in Physiology or Medicine in 2015.

Since it was first isolated, in 1972, several artemisinin analogs with improved solubi-lity and pharmacokinetic profiles have been developed [39]. These include dihydroartemisinin (DHA), artemether, arteether, and, especially, artesunate, which is a hemisuccinate ester of DHA. Artesunate because of its substantial water solubility and high oral bioavailability is considered superior to artemisinin and other derivatives that are lipid-soluble [40]. In a relatively recent study based on computer simulations and pharmacokine-tics, the oral bioavailability of artemisinin was found to be 12.2 ± 0.832% [41], since the oral bioavailability of artesunate, which shows high variability, can reach 60% [42,43]. DHA, which is the common active metabolite of artemisinin derivatives, into which they are converted in the body upon administration, but it is also available as an independent drug, has an even higher bioavailability of >80% after oral administration [44]. However, all artemisinins have very short in vivo half-lives, ranging from 2 to 5 h for artemisinin, <1 h for artesunate and DHA and 2 to 4 h for artemether [43,44]. Therefore, they are commonly coadministered antimalarial agents with longer half-lives. These combinations also help to slow the development of parasite resistance [45,46]. The fast elimination of artemisinins also explains, at least partially, their favorable safety profiles. Furthermore, because of their ability to easily cross the BBB, artemi-sinins are highly effective in the treatment of the cerebral form of the disease [42].

All forms of the drug encompass a tetracyclic core bearing an endoperoxide bridge (C-O-O-C), which is essential for the antimalarial activity of these lactons. For this, artemisinins are thought to be activated through an interaction with intraparasitic heme in erythrocytes—a byproduct of hemoglobin endocytosis and catabolism within the malaria parasite—resulting in heme iron (II) oxide-mediated cleavage of the endoperoxide bridge. The subsequently generated carbon-centered free radicals then alkylate and damage susceptible cellular proteins, lipids and other molecules, as well as generate reactive oxygen species (ROS), resulting in the death of the parasite [47]. Additionally, there is evidence suggesting that DHA can kill malaria parasites, inducing proteostatic stress by compromising parasite proteasome function, at which time the accumulation of unfolded/da-maged and polyubiquitinated proteins will activate the ER stress responses [48].

Presently, extensive in vitro and in vivo preclinical data support the therapeutic efficacy of artemisinins in a variety of human disease conditions beyond malaria, such as cancer, diabetes, atherosclerosis, viral infections, autoimmune diseases and, not least, in neurodegenerative diseases [49]. These effects seem to be mediated by artemisinins-induced changes in several signaling pathways, that interfere with multiple hallmarks of the respective diseases and probably rely on multitarget and promiscuous interactions of artemisinins with cellular proteins [50]. However, the exact molecules and substances that directly interact with artemisinins have yet to be determined.

## 3. Alzheimer’s Disease Mouse Models Used for Testing Artemisinins

Wild-type mice do not develop Aβ plaques during the course of normal aging, thus the most commonly used animal models for AD are transgenic mice that rely on the neural overexpression of human genes carrying mutations associated with familial AD (FAD). It was observed that the expression of multiple FAD-associated mutations at one time results in transgenic mice with a more severe pathology that develops at a younger age [51]. Thus, a vast majority of transgenic mice express mutated human APP in combination with PSEN1, resulting in increased Aβ production, Aβ aggregation and formation of amyloid plaques with or without human MAPT (microtubule-associated protein tau), which results in the formation of neurofibrillary tangles [52]. It is important to emphasize that none of the available animal models replicate all features of human AD; they only recapitulate one or more features of AD pathology, commonly in a nonphysiological manner, which represents their major limitation. In addition, the exact phenotype, including the type and time course of the AD pathology, strongly depends on the FAD mutation, promoter used, expression levels of transgene in the brain and, not least, the background mouse strain, thus making absolute comparisons among models difficult [51]. Nevertheless, the results ge-nerated using experimental models have contributed substantially to the elucidation of the molecular pathogenesis of AD and development of diagnostic biomarkers and therapeutic strategies, although in the latter with a very high rate of failure when tested in clinical settings. This has led some scientists to question the validity of the available mouse models for developing therapeutic strategies in humans [51,53]. The contrary is supported by the monoclonal antibodies targeting of different forms of Aβ recently approved by the US Food and Drug Administration (FDA) for the treatment of AD. Prior to clinical trials, these antibodies were tested in several preclinical studies decreasing pathogenic Aβ levels and preventing Aβ deposition in the brain of transgenic mice [54,55]. Furthermore, the use of more than one model to evaluate potential drugs should enhance the chances of a successful translation from preclinical studies to patient therapy. Here, we summarize the main characteristics of AD mouse models used by the different research groups to test the effects of artemisinins on various aspects of the disease.

APP_swe_PS1_L166P_—APP/PS1 mice are double transgenic mice coexpressing the KM670/671NL “Swedish”-mutated amyloid precursor protein (APP) and the L166P-mutation carrying human presenilin 1 (PS1) under the control of a neuron-specific Thy1 promoter element. The Aβ_40_ and Aβ_42_ concentrations increase with advancing age in the brain of APP/PS1 mice. Amyloid plaque deposition starts at approximately 6 weeks of age in the neocortex and 3–4 months of age in the hippocampus in parallel to plaque-associated neuroinflammation (microgliosis and astrogliosis), similar to that in human AD. Cognitive impairment, including deficits in the Morris Water maze, was reported at seven months of age. The major limitations of APP/PS1 mice are a lack of widespread neuronal loss and neurofibrillary tangles (NFTs), yet they show increased tau hyperphosphorylation [56,57].

APPswe/PSEN1dE9—APP/PS1 mice represent the most widely used AD model and were bred by crossing transgenic APP animals expressing the Swedish mutation with those expressing PSEN1dE9 (PSEN1 gene without exon 9). In comparison to the APP_swe_PS1_L166P_ model, these mice start developing Aβ deposits later, by six months of age, showing abundant plaques in the hippocampus and cortex by 9 months, with further increases up to 12 months of age [58,59]. In parallel, astrocytosis develops, indicated by extensive GFAP staining throughout the cortex by 15 months [60]. Modest neuronal loss was observed adjacent to plaques between 8 and 10 months of age [61] but tangles were not detected in this model.

The 5xFAD mouse model carries five FAD mutations, specifically the Swedish (APP_K670N/M671L_), London (APP_V717I_) and Florida (APP_I716V_) APP mutations and the PS1_M146L_ and PS1_L286V_ mutations, driven by the Thy-1 promoter [62]. In these mice, a very early intraneuronal Aβ accumulation (6 weeks) is followed by plaque formation at 2 months, when astrogliosis and microgliosis also begin to develop. Soluble Aβ_42_ is already detectable at 1.5 months. The Aβ_42_/Aβ_40_ ratios are very high in young mice, averaging ∼25, but they decrease to ∼5 as a result of the increase in Aβ_40_ levels with age. The mice exhibit synaptic degeneration and neuronal loss, altered spatial working memory, and develop progressive cognitive deficits as early as 4–5 months of age. However, abnormal tau hyperphosphorylation was not noticed in the 5XFAD mice, and they also failed to develop NFTs. The early onset and aggressive amyloidosis render this moue model for the study of, specifically, AD-like amyloidosis and the effects of plaques on the brain and testing potential therapies on it [62].

The 3xTg mouse model is considered the most complete transgenic mouse model of AD pathology available that combines the tau (MAPT) mutation P301L with the APP Swedish mutation and PSEN1 mutation M146V. The APP and MAPT expressions are driven by the Thy-1.2 promoter, while PSEN1 expression is driven by the endogenous PSEN1 promoter [63]. These mice develop a progressive neuropathology, including intracellular (3–4 months of age) and extracellular Aβ deposits (6 months of age) and phosphorylated tau aggregates (10–12 months of age), which start in the hippocampus and then expanding to the neocortex, thus closely mimicking the development pattern of the pathology in the brain of humans with AD [64]. Gliosis may occur earlier than 7 months [65]. Mice also show minor, localized neuronal cell loss, synaptic impairment and cognitive deficits from 4 months, when plaques and tangles are not yet apparent [51]. Interestingly, recent reports indicate that, since the original report, in 2003 [63], the development of pathology has been substantially delayed (~18 months of age), and a sex difference manifested for both plaques and tangles, only emerging in female mice [66]. The model is considered suitable for assessing relationships between amyloid and tau pathologies, as well as cognition and testing of potential therapies [67].

Intracerebral injections of Aβ oligomers: injection or infusion of soluble Aβ peptide into the brain is considered an alternative to transgenic animals to mimic AD pathology in physiologically normal, nontransgenic mice or rats and to study the effects of increased soluble amyloid species in the brain without the presence of plaque. The results appear to depend on the site of injection and the nature of the peptide used [68]. The injection of Aβ_1–42_ into the rat\mouse hippocampus was able to induce memory deficits and increase the level of oxidative stress and inflammatory response and even apoptosis of cholinergic neurons, indicating that these phenomena are general consequences of Aβ_1–42_ administration in the brain. Additionally, phosphorylated tau proteins and some neurofibrillary tangles were also observed in the hippocampus of these rodents [68]. The model is considered a valid tool to evaluate the potential for compounds to directly target Aβ or its downstream mechanism early in AD.

## 4. Effects of Artemisinins on Hallmarks of AD Pathogenesis

**Figure 1:**
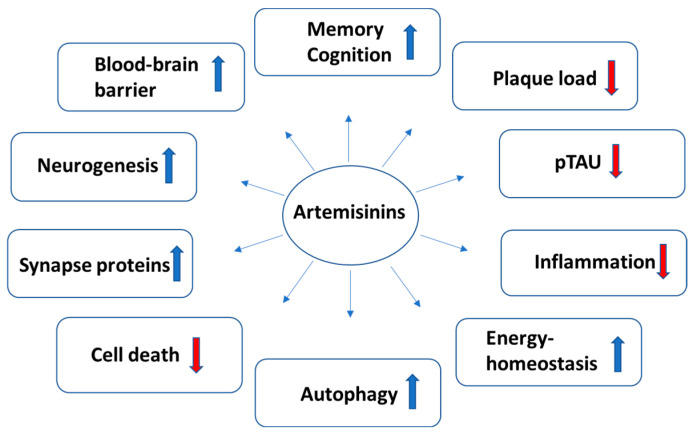
Reported effects of artemisinins on the hallmarks of Alzheimer’s disease. The arrows indicate changes induced by artemisinins in the specified pathogenetic factors and hallmarks of AD in preclinical studies; ↑ (increase) and ↓ (decrease) compared to AD condition.

The reported effects of artemisinins on hallmarks of AD, observed using in vitro and in vivo preclinical models, are summarized in Figure 1.

### 4.1. Aβ Pathology—Plaque Formation

According to the amyloid hypothesis, the accumulation of pathological forms of Aβ represents the primary pathological process in AD pathogenesis, which is driven by an imbalance between Aβ production and Aβ clearance, resulting in the formation of amyloid plaques [1]. Aβ aggregation may disrupt cell-to-cell communication, and it was shown to induce neuroinflammation and, finally, neuronal cell death. Moreover, increasing evidence indicates that soluble Aβ oligomers and the intermediate products of APP cleavage by β-secretase (CTFs) could also trigger tau alterations and contribute to neurotoxicity and neurodegeneration [1,69], leading to cognitive decline. Correspondingly, extensive experimental data propose that the inhibition of Aβ generation and plaque formation should reduce Aβ pathology and attenuate tauopathy [8]. The new, recently FDA-approved antibody drugs (aducanumab, lecanemab and donanemab) for use in AD therapy targeting the formation of Aβ plaques have shown an ability to slow down cognitive decline in patients with early symptoms, strengthening the hypothesis of a causal role of Aβ in the pathogenesis of AD [70,71]. However, the magnitude of the clinical effect elicited by these drugs (an approximately 30% slower rate of decline) is rather modest, suggesting the contribution of additional pathological mechanisms to the disease.

In this context, it is important to note that several independent studies have reported a reduction in Aβ production and plaque load in the cortex and hippocampus of various AD mouse models upon treatment with different artemisinins. Already one decade ago, Shi et al. demonstrated that artemisinin treatment in 5-month-old APPswe/PS1dE9 transgenic mice at a dose of 40 mg/kg/day for 30 days decreased the neuritic plaque burden by approximately 48% and 61% in the cortex and hippocampus, respectively. Moreover, these authors have shown that artemisinin reduced APP processing through inhibition of the β-secretase activity, evidenced by decreased BACE1 levels in transgenic mice brains [72]. Very similar results were obtained when 6-month-old male APPswe/PSEN1dE9 mice were treated with DHA at a dose of 20 mg/kg/day. In this study, the oral administration of DHA for 3 months reduced thioflavin S-stained Aβ deposits and the quantities of amyloid plaques in the cortex and hippocampus sections of the treated group in parallel with markedly reduced Aβ_42_ levels in both the cortex and hippocampus homogenates. Compared with the control group, the levels of APP and BACE1 protein were also downregulated after DHA treatment, indicating reduced Aβ production [73]. In another study, 3-month-old male APPswePSEN1dE9/Nju mice were fed with relative high concentrations of DHA, specifically 50 mg/kg or 300 mg/kg, until they reached 9 months of age. Thioflavin T-staining of brain tissue sections revealed that both low and high doses of DHA significantly reduced Aβ plaque aggregations in the CA3, CA1 and DG subregions of the hippocampus and in the cortex of AD mice, importantly, without toxicity or side effects on mice livers and kidneys [74]. Interestingly, in this study glycine silver staining was used to detect NFTs, evidencing their significant reduction in the brains of DHA-treated mice. Further, using the same mouse model, Qin et al. reported that the semisynthetic derivative of artemisinin, artesunate, also alleviated AD phenotypes in these APP/PS1 mice, reducing Aβ deposition in brain sections, as well as the levels of soluble and insoluble Aβ_1–42_ in brain tissue, both by 40–50% [75]. In all of these studies, the treatment of APPswe/PSEN1dE9 mice corresponded mostly to the initial stage of Aβ deposition in this mouse model, supporting a preventive potential of artemisinins treatment in AD.

In line with this, very recently, artesunate was administered (32 mg/kg/day, i.p.) to 3-month-old 5xFAD mice that were also deficient in phosphatidylinositol-binding clathrin assembly protein PICALM (*Picalm*^+/−^; *5XFAD* mice), a protein involved in the regulation of endocytosis and internalization of cell receptors, as well as intracellular protein trafficking [68]. After two months of treatment with artesunate, these mice exhibited relatively early-stage Aβ pathologies, two-fold increases in brain capillary PICALM levels and, in parallel, reduced Aβ_42_ and Aβ_40_ levels, Aβ load, and thioflavin S-positive amyloid deposition in the cortex and hippocampus of 34–51%. However, in this study, no differences in APP processing, as shown by comparable levels in the brain for APP, APP-CTF, BACE1 or Aβ degrading enzymes (neprilysin and insulin-degrading enzyme) were found [76].

In our own study, 12-month-old male APP_swe_/PS1_L166P_ mice, characterized with early onset amyloid plaque development, were evaluated after 3 months of oral administration of artemisinin or artesunate. The administration started at a relatively advanced stage of amyloidosis, and both drugs, at a dose of 10 mg/kg/day, reduced the plaque load in the cortex and hippocampus by ~40–50%, and a weaker effect or none at all was observed at a higher dose of 100 mg/kg/day [77]. In addition, artesunate treatment resulted in a significant decrease in APP cleavage products, specifically CTF levels and soluble Aβ concentrations in the hippocampus homogenates of 12-month-old APP/PS1 mice, altogether supporting that artemisinins, especially artesunate, can not only prevent but eventually reverse the progression of amyloid deposition in the brain of APP/PS1 mice. In conclusion, these different studies demonstrate convincingly that artemisinins treatment is an efficient method to reduce the level of amyloid plaques in AD models, although no complete elimination of amyloid plaques could be achieved in neither study. Furthermore, dose- and drug-dependent effects have to be considered.

### 4.2. Tau Pathology

Aβ pathology is thought to promote the development of tau pathology by favoring the conversion of tau from a normal to a toxic state that can even enhance Aβ toxicity via a feedback loop [78]. The reduction in cerebrospinal fluid and plasma p-tau by the recently approved anti-amyloid monoclonal antibodies in clinical trials support a downstream effect of amyloid-reducing agents on AD tau pathology as well [71]. Several studies using the 3xTg mouse model that exhibits both amyloid plaques and NFTs have shown that in addition to Aβ, artemisinins also beneficially modulate tau homeostasis.

Under normal physiological conditions, tau protein is a microtubule-associated protein highly expressed in the axons of neurons and involved in promoting the assembly and stabilization of microtubules. In AD, tau protein has been found in a hyperphospho-rylated state at certain serine/threonine residues forming aggregates of insoluble paired helical filaments and NFTs, which then lead to impairment of axonal transport compromising both neuronal and synaptic functions [79]. Interestingly, tau can also be secreted into the synaptic cleft in an activity-dependent manner, subsequently being internalized by a postsynaptic neuron or glia cells, spreading to other brain regions [80]. It was found that mostly phosphorylated soluble tau oligomers accumulate in both pre- and postsynaptic terminals, suggesting that mainly these tau forms spread trans-synaptically. These and other findings support a specific role for soluble pathological forms of tau in the neuropathology of AD [81].

Li et al. reported, in 2019, that artemether administered at doses of 5 mg/kg or 20 mg/kg, reduced both the Aβ deposition and phosphorylation of tau (Ser 416) by 20–40% in the brain cortex of 10-month-old 3xTg AD mice after treatment for four weeks [82]. In another study, the beneficial effects of artemether against Aβ_25–35_-induced cognitive impairments in a rat model were correlated with the downregulation of the endogenous expressions of Aβ, BACE1, mTOR and tau proteins in N2a cells [83]. Artemisinin was also tested in 3xTg mice. Treatment for 1 month at doses of 1, 5 or 10 mg/kg/day for one month resulted in a significant reduction in with Aβ antibodies and Congo red staining detected Aβ levels and plaque load in the cortex and hippocampus of 12-month-old 3xTg mice. Additionally, significant reductions in the phosphorylated tau (Ser396 and Thr212) levels but not in total tau levels were recorded in the brains of artemisinin-treated mice when compared to control 3xTg mice [84]. These results were confirmed by a recently published study using Artemisia annua extracts in different concentration rather than isolated artemisinins [85]. In this study, the oral administration of the extract (6.7 mg/mL vs. 20 mg/mL) to 9-month-old female 3xTg AD mice for 3 months reduced both Aβ accumulation and tau hyper-phosphorylation (Thr181) to within a comparable range in the cortex and hippocampus. Interestingly, similar to our own observations, some clear dose dependent effects of the extract were evidenced. The effect of extract on Aβ deposition and APP and Aβ_1–42_ levels in brain homogenates was higher in animals treated with lower concentrations of the extract since mice receiving the dosage of 20 mg/mL exhibited a more prominent reduction in tau-phosphorylation compared to animals treated with 6.7 mg/mL. The plant extract did not affect the expression of total tau, altogether indicating an eventual direct effect of the artemisinin extract on tau pathology [85].

In conclusion, several independent studies convincingly demonstrate that artemisinins treatment is an efficient method to reduce the level of Aβ and amyloid plaques, as well as tau hyperphosphorylation, in different AD mouse models, mimicking different stages of the disease, although the range of these reductions did not exceed 40–50% in either study.

### 4.3. Inflammation

Inflammation is another central feature of AD pathology, defined mainly by activation of CNS-residing glial cells and release of inflammatory factors. Inflammatory biomarkers are elevated in AD patients [86], and glial cells, such as microglia and astrocytes, that surround senile plaques and affected neurons are usually observed in the brain of AD patients [87]. Data from genome-wide association studies (GWASs) on sporadic AD cases showing associations between AD and genes involved in innate immunity (e.g., TREM2, CD33) suggest that inflammation is probably not only a consequence of the accumulation of Aβ and p-tau in the AD brain but may also modulate disease progression [87,88]. Both microglia and astrocytes can adopt neuroprotective and neurotoxic phenotypes, which seem primarily related to the stage of the disease. Microglia are the primary immune cells of the CNS and thought to protect against the incidence of AD due to the clearance of Aβ aggregates. Indeed, the ability of microglia to prevent plaque development by removing pathogenic Aβ is part of a first-line defense in addition to the inhibition of tau hyperphosphorylation and the production and release of neurotrophic factors. On the other hand, the Aβ-mediated activation of these cells can cause the release of pro-inflammatory cytokines, such as tumor necrosis factor (TNF)-α, interleukin (IL)-1β, and IL-6, and induce the generation of neurotoxic factors, such as nitric oxide and ROS, resulting in neuronal damage [89]. Aβ- or microglia-activated astrocytes also have neuroprotective functions in AD, ensuring the functional integrity of neurons and synapses with the release of neurotrophic factors and by blunting plaque formation by Aβ clearance and maintenance of BBB integrity. However, they may also favor neuroinflammation through the delivery of inflammatory cytokines and chemokines [87]. Pro-inflammatory cytokines can then upregulate β- and γ-secretases, generating a self-sustaining cycle in which cytokines and Aβ load reciprocally increase. Cytokines, especially IL-6, stimulating CDK5 activation promote the hyperphosphorylation of tau, indicating that long-lasting neuroinflammation can also exacerbate Aβ and tau pathologies in AD [90,91]. Thus, modulation of neuroglial activation may represent another effective intervention to control the pathophysiology of neurodegenerative diseases [92].

Aβ-activated glial cells’ production of different pro-inflammatory cytokines involves NF-κB activation that also favors the stimulation of NLRP3 inflammasome signaling cascades, resulting in the activation of caspase-1 and the secretion of IL-1β and IL-18 [93,94]. Thus, molecules that influence NF-kB activity can indirectly alter NALP3 priming. The involvement of this mechanism in the pathogenesis of AD is supported by the finding that the inhibition of NLAP3 in in vivo AD models can rescue cognitive deficits.

A large number of studies provide evidence for the anti-inflammatory activities of artemisinins in AD mouse brain. In 2013, Shi et al. had already reported that artemisinin treatment in 5-month-old APPswe/PS1dE9 transgenic mice at a dose of 40 mg/kg/day for 30 days reduced the nuclear translocation of NF-κB p65 and increased I-κBα expression, which led to the inhibition of NF-κB activity and a decrease in the production of downstream cytokines (IL-6 and TNF-α) of 37.16% and 34.46% in transgenic mice brains [72]. Additionally, in the artemisinin-treated mice, the level of NALP3 significantly decreased by 37%, and the level of the downstream molecule caspase-1 p20 subunit and the production of IL-1β decreased by 50% and 29.73%, respectively. Thus, artemisinin was shown to inhibit NF-κB activity and NALP3 activation in APPswe/PS1dE9 double-transgenic mice in a relative early stage of AD pathogenesis, preventing tissue damage. More recently, in the same mouse model artesunate administration from 2 to 6 month of age at either a 5 or 10 mg/kg/day dose effectively reduced the expressions of TNF-α, IL-6, and IL-1β and also reversed the alterations in the mitochondrial dynamics and mitophagy in mouse brain tissues [75]. Correspondingly, in the same study, artesunate suppressed Aβ-induced activation of BV-2 microglial cells and N2a neuronal cells reducing the secretion of TNF-α, IL-6, and IL-1β (protein and mRNA levels) to 100 and 200 nM but not 50 nM concentrations. In a very similar fashion, Artemisia annua extract was recently shown to attenuate neuroinflammation in the brain of 12-month-old female 3xTg mice after 3 months of treatment. GFAP and Iba-1 (a microglia marker) expressions, as well as levels of IL-6, TNF-α, and IL-1β, were significantly reduced in the hippocampus and cortex of the mice treated with either 6.7 mg/mL or 20 mg/mL, in parallel exhibiting lower Aβ accumulation, tau hyperphosphorylation, and milder cognitive deficits [85]. These results are supported by findings in nontransgenic mouse AD models. In a study by Qiang et al., male KM mice, pretreated for 2 weeks by intragastric administration of artemisinin B (20, 40 or 80 mg/kg), were injected via the lateral ventricle with Aβ_25–35_ and treated for an additional week postsurgery, resulting in increased levels of anti-inflammatory IL-10 and reduced amounts of pro-inflammatory TNF-α in the cortex and hippocampus, as well suppressing Iba1-positive cell activation in the hippocampal CA1 region [95]. In a more recent study, six-week-old C57 mice, pretreated for one month with intraperitoneal administration of artemisinin at 5 mg/kg/day, were injected with Aβ_1–42_ into the hippocampus. The analysis of the hippocampi at day 7 postsurgery revealed that artemisinin treatment significantly reduced the number of GFAP- and Iba1-positive cells, as well as the release of the inflammatory factors TNF-α, IL-1β and IL-6. Moreover, the protein levels of NF-κB, p65 and Toll-like receptor 4 (TLR4) also decreased, supporting earlier findings that the anti-inflammatory effect of artemisinins may be achieved by suppressing the NF-κB signaling pathway [96]. Further support for the anti-inflammatory effect of artemisinins in AD models was provided by a current publication showing that artesunate (32 mg/kg/day, i.p.) also suppressed the neuroinflammatory response in the hippocampus and cortex of *Picalm*^+/−^; *5XFAD* mice, as indicated by the detection of significantly lower numbers of Iba1-positive microglia (by 28–25%) and GFAP-positive astrocytes (by 23–36%) [76].

In conclusion, several studies convincingly support a strong anti-inflammatory function of different artemisinin derivatives in AD models, which is in agreement with many other works that describe the anti-inflammatory potential of artemisinins in another context [33].

### 4.4. Oxidative Stress and Mitochondrial Dysfunction

The overproduction of ROS in combination with insufficient antioxidant defense leads to cellular oxidative stress. Markers of oxidative stress, including high levels of oxidatively modified nucleic acids, lipids, and proteins, were found in postmortem brain tissue and biological fluids from patients with preclinical or early stages of AD and ApoE4 (apolipoprotein E epsilon-4) carriers, as well as in animal AD models. Moreover, in AD, the levels of antioxidant enzymes were also altered [97]. Among the multiple factors that can contribute to ROS production in AD brains, such as neuroinflammation and abnormal metal homeostasis (Fe, Cu and Zn), mitochondria are the major source of ROS generation [98]. It is thought that Aβ oligomers trigger a significant influx of Ca^2+^ into the mitochondria, causing membrane depolarization, enhanced ROS production, metabolic dysfunction, and eventually cytochrome C release, initiating apoptosis [99]. The evidence of altered energy and oxygen metabolisms and mitochondrial dysfunction early in AD pathogenesis, before any sign of Aβ or tau pathology, led to the mitochondrial cascade hypothesis proposing that the aggregations of Aβ and tau in AD might be a compensatory response to underlying oxidative stress [100]. Either way, once initiated, a “vicious cycle” may be created in which dysfunction of mitochondria contributes to disease progression directly affecting synaptic activity and neurotransmission, leading to cognitive deficits [101]. Nevertheless, supplementation with antioxidants that target total ROS in the orga-nism (e.g., vitamins E and C and α-lipoic acid) has shown a small modifying effect on AD development in clinical trials. This may rely, at least partially, on the dual role of ROS, implying that in addition to their implication in neurodegeneration, some oxidant species, such as superoxide and hydrogen peroxide (O_2_^2−^ and H_2_O_2_) function as signaling molecules of essential redox-dependent signaling pathways [101]. Thus, antioxidants that directly target mitochondria seem to represent a more efficient approach to attenuating local ROS production, and some of these compounds have already led to promising results in clinical trials [102].

A number of different studies indicate that artemisinins alleviate oxidative stress and mitochondrial damage in in vivo and in vitro models of AD. Li et al. reported, in 2019, that artemether attenuated oxidative stress in the brain cortex of 10-month-old 3xTg-AD mice. After artemether treatment at a dose of 20 mg/kg but not at 5 mg/kg, the levels of the lipid peroxidation product MDA (malondialdehyde) and antioxidant enzyme SOD (superoxide dismutase) reversed by approximately 25% in brain extracts of 3xTg-AD mice in comparison to untreated AD mice [82]. Similar findings were reported in a rat model of streptozotocin-induced AD and diabetes after the administration of 50mg/kg artemisinin for 4 weeks [103]. Furthermore, it was demonstrated that artemether stimulated AMPK/GSK3β(Ser9)/Nrf2 signaling, resulting in an increased level of the antioxidant protein heme oxygenase-1 (HO-1), both in vivo and in vitro, in A*β*_1–42_-treated PC12 cell cultures, correlating with a reduction in neuronal cell death [82]. Notably, transcription factor nuclear factor erythroid-2-related factor 2 (Nrf2) is generally activated in cells as an additional mechanism to protect against oxidative stress activating expressions of cytoprotective genes [104]. Additionally, it was shown that pretreatment with 10–100 μM of artemether in PC12 cells diminished also the A*β*_1–42_ induced decline in mitochondrial membrane potential. Similar findings, of reduced ROS levels and restored mitochondrial membrane potential attenuating apoptosis after exposure to Aβ_1–42_ were reported for artemisinin in SH-SY5Y human neuroblastoma cells [73] and for both artemisinin (0.25–1 μM) and artesunate (100 and 200 nM) in BV2 microglial cells [75,96]. Remarkably, artesunate was shown, not only to recover the depolarization of mitochondrial membranes suppressing A*β* induced oxidative stress but also to regulate mitochondrial dynamics in BV-2 and N2a neuronal cells [75].

Coordinated cycles of fission and fusion are essential for maintaining mitochondrial morphology and functions and requires the adequate expression of genes coding proteins such as dynamic-related protein-1 (Drp-1), mitochondrial fission-1 (Fis-1), fusion proteins (Mfn1, Mfn2 and Opa1), that are disturbed by both Aβ and CTFs already in early-stage AD, resulting in mitochondrial fragmentation [105,106]. To maintain mitochondrial homeostasis, damaged mitochondria regularly undergo mitophagy, involving the engulfment of altered mitochondria by the autophagic vacuoles and their fusion with lysosomes, followed by subsequent degradation and recycling. In both, AD patients and animal models, an age-dependent accumulation of defective mitochondria and impaired mitophagy were coupled with increase in oxidative stress and dysfunctional neurons, whereas mitophagy stimulation improves memory impairments [107,108]. Qin et al. recently demonstrated that artesunate antagonized the effects of Aβ on mitochondria dynamics proteins (Drp-1, Fis-1, Mfn-1 and Opa-1) and reversed the suppression of autophagy and mito-phagy related proteins induced by Aβ in BV-2 and N2a cells as well as in APPswe/PS1dE9 double transgenic mice [75]. Thus, several studies on different models strongly suggest that the artemisinins’ neuroprotective effects in AD are mediated in part by their antioxidant activities including reduction in pro-oxidant molecules and increasing production of endogenous antioxidants as well as regulating mitochondrial dynamics and mitophagy. However, the beneficial effects of artemisinins treatment on mitochondrial structure and function seem to be strongly concentration- and context-dependent, since higher concentrations of artesunate (20 μM to 40 μM) were found to inhibit mitochondrial respiration in cancer cells, resulting in oxidative stress and cell damage [109].

### 4.5. Autophagy

Autophagy is the process by which not only damaged cellular organelles but also protein aggregates and cellular debris are degraded via lysosomes, thus representing an important cellular defense mechanism for maintaining cellular homeostasis. The autophagic process comprises several steps, including sequestration, fusion and degradation, and its dysregulation has been observed in AD patients’ brains and animal models [110,111]. The increased numbers of autophagosomes and autolysosomes observed in the brains of patients with AD are probably caused, on the one hand, by the activation of autophagy and, on the other hand, the reduction in degradation by autolysosomes. Specifically, hyperactivation of mTORC1 (mammalian target of rapamycin complex 1) signaling was demonstrated in AD, which may prevent autophagy initiation and autophagosome formation. Consequently, the enhancement of autophagy to remove Aβ, CTFβ and p-tau proteins, preventing cell death, may be a promising therapeutic strategy, especially in the early stages of AD [111,112]. In this context, a study by Zhao et al. has convincingly demonstrated that DHA, the active metabolite of artemisinins corrected autophagy dysfunction in the initial stage of Aβ pathology in APPswe/PSEN1dE9 mice and in cell models of AD (N2a-APP and SH-SY5Y-APP) by acting on multiple targets within the autophagic process [99]. Transmission electron microscopy and measurements of autophagy stage-specific proteins have evidenced that DHA treatment not only activates autophagy via the upregulation of ATG5, ATG12, ATG16L and LC3 II/I and decrease in ubiquitin-binding p62 protein levels, but it also promoted the fusion of autophagosomes and lysosomes (increases in Beclin1, ATG14, Rab7 and RILP levels) and, thus, elevated the number of lysosomes and their degradation function. Moreover, their findings indicated that DHA can activate autophagy by suppressing the mTOR/ ULK1 (unc-51-like kinase) junction within the autophagy-regulating signaling network [113].

### 4.6. Cell Death

Neuronal loss is one of the main features of AD, but it is difficult to detect in real time because of the limited accessibility of dying cells by immunohistochemical methods [114]. Loss of neurons in AD begins in the preclinical stage of the disease and progresses during MCI and dementia, correlating well with the degree of cognitive deficits. Multiple sources of evidence indicate that different forms of cell death can occur in AD, including apoptosis, necroptosis, pyroptosis, autophagic cell death, ferroptosis and necrosis in different stages of the disease [114]. However, it is not yet clear which pathway is dominant in the different stages of the disease and/or whether there is a combinatorial effect among coactivated death pathways [115].

The features of apoptosis, a type of regulated cell death, include cytoplasmic shrinkage, blebbing of the plasma membrane, nuclear condensation and fragmentation, and the formation of apoptotic bodies can be observed in neurons incubated with Aβ peptide and AD brain tissue [114]. Apoptosis can be triggered by two distinct pathways: the intrinsic (also called the mitochondrial or Bcl-2-regulated) pathway and the extrinsic (also called the death receptor) pathway, with the latter precipitated by the binding of extracellular death ligands, such as TNF-α, FasL, and TRAIL (tumor necrosis factor (TNF)-related apoptosis-inducing ligand), to transmembrane death receptors. In the intrinsic pathway, mitochondrial membrane depolarization, as a result of cellular injury, such as DNA damage, metabolic stress, and ROS, leads to the release of cytochrome C from the mitochondria, resulting in the activation of effector caspases and apoptotic death. Proteolytic enzymes, known as caspases, are involved in both pathways. Moreover, the family of B-cell lymphoma-2 (Bcl-2) proteins, including pro-apoptotic and anti-apoptotic members, were identified as essential integrators of signals that trigger cell survival or apoptosis [116]. TUNEL staining, the in situ labeling technique for apoptotic cell detection, and the levels of caspase 3, the main executive caspase functioning in multiple apoptotic signaling pathways, have been reported as elevated in cortical and hippocampal neurons from AD patients. In addition, the expressions of several pro- and anti-apoptotic members of the Bcl-2 family, such as Bcl-2, Bcl-xl, Bax, and Bak, are altered in AD brain extracts, including humans and mouse models [114].

The effects of artemisinins on apoptotic cell death were studied in different models of AD, and the results indicated clearly anti-apoptotic properties of artemisinins. Li et al. reported, in 2019, a reduced number of TUNEL-positive apoptotic neurons in the brain cortex of 10-month-old 3xTg-AD mice following treatment with 5 mg/kg or 20 mg/kg of artemether and in different Aβ_1–42_-exposed neuronal cell cultures (PC12, SH-SY5Y, mice primary cortical neurons) pretreated with artemether (10–100 μM), concomitant with decreased caspase 3 activity. Moreover, the expression of the anti-apoptotic protein Bcl-2 increased, whereas the pro-apoptotic protein Bax decreased in brain homogenates of treated 3xTg AD mice. These authors linked the inhibition of brain cortical apoptosis and attenuation of cognitive deficits in the 3xTg mice model to AMPK/GSK3*β*(Ser9)/Nrf2 activation and consecutively increased antioxidant effects of artemether [82]. In addition, Zhao et al. observed, in parallel with attenuated histopathological changes, a reduction in the number of apoptotic cortical neurons in 12-month-old 3xTg-AD mice after one month of treatment with artemisinin (1, 5 or 10 mg/kg). Cytochrome C, caspase 9 and caspase 3 activities decreased, as the ratio of Bcl-2/Bax increased in response to the artemisinin treatment, both in the 3xTg mice and the Aβ_1–42_-induced SH-SY5Y cells (12.5 μM). The rise in the expression levels of p-ERK1/2 and p-CREB detected both in vivo and in vitro suggested that artemisinin reduced brain neuronal apoptosis through the ERK/p-CREB/Bcl-2 axis [117]. These results are in concordance with a much earlier in vitro study describing the protective effect of 25 μM artemisinin against Aβ_25–35_-induced cell death in neuronal PC12 cells through activation of the ERK1/2 pathway [118]. Moreover, it was shown that artemisinin (5 mg/kg/day) reduced apoptotic cell death in the cerebral cortex of C57 mice after intrahippocampal injection with Aβ_1–42_. Additionally, these authors found that artemisinin-pretreated microglia medium reduced PC12 cell apoptosis and increased PC12 cell viability, demonstrating that artemisinin protects against neuronal cell apoptosis by improving the inflammatory environment [96]. Accordingly, in cocultures of Aβ-pretreated microglial BV-2 and neuronal N2a cells, when BV2 cells were pretreated with both Aβ and artesunate they failed to induce apoptosis of N2a cells [75]. Consistently, DHA (20 mg/kg/day), the active metabolite of all artemisinin compounds, rescued neuronal loss in the hippocampal CA1 area of 9-month-old APPswe/PSEN1dE9 mice and downregulated the protein expression levels of full-length caspase 3, cleaved caspase 3 and Bax while also increasing the level of the anti-apoptotic Bcl-2 protein. Concurrently, DHA corrected the abnormal levels of brain-derived neurotrophic factor (BDNF) [117]. More recently, an artemisia extract was reported to rescue neuronal cell apoptosis in 9-month-old 3xTg AD mice accompanied by changes in the expressions of different apoptosis regulators. In addition to the emodulation of Bax, Bcl-2 and cleaved caspase 3 levels, artemisinin extract treatment activated YAP/TEAD2/Survivin signaling, promoting a significant increase in anti-apoptotic Survivin protein levels, while significantly reducing the level of pro-apoptotic promyelocytic leukemia protein [85]. YAP (Yes-associated protein) is a transcriptional cofactor that regulates cell death and survival by binding to different transcription factors such as p73 and TEAD (TEA-domain family member) and seems to be a key player in the molecular network of AD [119]. Since a YAP-mediated increase in the TEAD activity has been implicated in cell proliferation, differentiation, and survival, reduced TEAD activity is thought to primarily promote necrotic cell death [120]. Interestingly, recent findings on MCI vs. AD patients and early- vs. late-stage 5xFAD mice strongly support that intracellular Aβ-triggered Hippo/YAP pathway-dependent necrosis occurs from the very early presymptomatic to late stages in both human and mouse AD pathologies. Moreover, residual Aβ after neuronal necrosis seems to be the seed for the formation of extracellular beta-amyloid plaques. Thus, early-stage interventions for molecules involved in the regulation of Hippo pathway-dependent apoptosis and necrosis could suppress progression of late-stage AD pathological changes, possibly including the limitation of extracellular Aβ aggregation [120].

Traditionally, necrosis is considered an unregulated form of cell death characterized by cell and organelle swelling, loss of membrane integrity and release of intracellular contents into the extracellular environment, usually triggering an inflammatory response. A main ultrastructural feature of intracellular Aβ-induced YAP-deprivation-mediated Hippo pathway-dependent necrosis is endoplasmic reticulum (ER) ballooning [121]. In a recent study, Zhao et al. reported that TEM analysis of neurons in brain sections of DHA-treated APPswe/PSEN1dE9 mice showed significantly reduced swelling of the ER [118]. Other forms of cell death acting in different stages of AD pathogenesis might also represents targets of artemisinins. Necroptosis, the regulated form of necrosis, exhibits the morphological features of necrosis but is associated with the activation of the RIP kinase cascade and the formation of the necrosome, and it can be induced by the stimulation of TNFR1, TLRs and certain other receptors [122]. Necroptosis has been primarily implicated in neuronal loss in the later stages of AD when they are exposed to amyloid plaques and tau tangles, possibly involving TNF-α inflammatory pathway signaling [123,124]. Pyro-ptosis is another form of necrotic programmed cell death involving the activation of caspase-1 by inflammasomes and the release of various inflammatory cytokines such as IL-1β and IL-18. The occurrence of pyroptosis (accompanied by inflammasome activation and elevated levels of IL-1β and IL-18) has been documented for many neurodegenerative diseases, including AD [125]. Ferroptosis refers to a form of iron-dependent necrotic programmed cell death that involves the accumulation of ROS and lipid peroxidation products and the depletion of reduced glutathione, caused by an imbalance in cellular redox homeostasis [126]. The specific role of ferroptosis in an AD setting is difficult to establish because of the overall presence of lipid peroxidation, altered iron homoeostasis and reduced glutathione in neurodegeneration; however, ferroptosis inhibitors manifested protection in animal models of AD and in clinical trials [127].

Altogether, a handful of different in vivo and in vitro data convincingly demonstrate that artemisinins protect directly against Aβ-dependent apoptotic neuronal cell death by regulating the expression of pro- and anti-apoptotic proteins and the activity of molecules involved in multiple intersecting pathways related to cell survival and cell death. In addition, the improved mitochondrial function and energy metabolism and diminished oxidative stress, as well as reduced neuroinflammation, by artemisinins, as outlined above, can presumably protect indirectly against apoptosis, necrosis and different forms of programmed necrotic cell death. Autophagy activation and autophagy flux correction can also play important roles in maintaining cell viability in the AD brain via artemisinins.

It is notable that in another context the cellular response to exposure to artemisinin and its derivatives include oxidative stress, DNA damage and repair, and induction of various modes of cell death [128]. Interestingly, artesunate was used as a ferroptosis inducer to selectively promote ROS- and lysosomal-iron-dependent cell death in KRAS-transformed PDAC cells, a cancer of the exocrine pancreas, while it exerted no effect on nonneoplastic human pancreatic ductal epithelial cells, further underscoring the multifa-ceted intervention potential of artemisinins [129].

### 4.7. Synapse Pathology

Both the direct effects of Aβ and tau on synaptic integrity and the indirect effects, through processes such as inflammation and mitochondrial dyshomeostasis, are likely to drive synaptic dysfunction and loss in AD [130,131]. Synapse dysfunction is thought to start long before the loss of memory and accelerate as the disease progress. There are widespread changes in synapse number, size, shape and structure of synaptic protein expression in AD brains, all suggestive of synaptic dysfunction that, predictably, will lead to changes in network oscillations [132,133]. There is evidence of a disrupted balance between excitatory and inhibitory neuronal activities (E/I imbalance) early before the onset of clinical symptoms, both in AD patients and animal models, and is seen as a main driver of AD pathogenesis promoting cognitive deficits [134,135]. It is well established that changes in excitatory synaptic transmission contribute to the E/I imbalance and AD progression due to aberrant activation of glutamate receptors, resulting in glutamatergic and cholinergic neuronal hyperexcitability and degeneration. Meanwhile, there is significant evidence suggesting that disruption to the inhibitory GABAergic synaptic transmission is also important and occurs early in the disease process [10,135,136,137,138]. Preclinical and clinical studies support that modulation of the GABAergic system may improve E/I imbalance and AD pathology, proposing it as a target for AD therapy [139].

In this context, it is interesting to note that several studies reported a significant impact of artemisinins on different components of the GABAergic system. In pancreatic islet cells, artemisinins were reported to increase GABAergic signaling resulting in elevated insulin secretion [140]. In contrast, studies using cultured spinal cord and hippocampal neurons described a decrease in glycinergic signaling and reductions in gephyrin and GABA_A_R coclustering [141]. These effects were found to be dependent on gephyrin, the main scaffold protein of inhibitory synapses, identified as a direct target of artemisinins. In particular, it was demonstrated that artemisinins bind competitively to the GlyRβ and GABA_A_ receptor-anchoring pockets of gephyrin. Moreover, in an additional study, it was shown that artemisinins bind to and inhibit the enzyme pyridoxal kinase, synthesizing pyridoxal 5-phosphate, an essential cofactor of glutamic acid decarboxylase (GAD) involved in the synthesis of GABA, with the therapy, thus, resulting in reduced inhibitory neurotransmission in vitro [142]. In comparison to these nonamyloidogenic in vitro experiments, in our own in vivo studies, artemisinin and artesunate, at two different doses (10 mg/kg and 100 mg/kg), rescued the expression levels of key proteins of inhibitory synapses, such as gephyrin and GABA_A_R-γ2, to approximately WT levels in the hippocampus of 12-month-old APP/PS1 (APP_Sw_PSEN1_L166P_), whereas in untreated APP/PS1 mice a robust reduction in synaptic protein levels and the number of synapses were detected [77,136]. Moreover, artemisinin, at a lower dose (10 mg/kg), was found to increase gephyrin protein levels, as well as the phosphorylation of gephyrin at serine 270 already in a preplaque stage of the disease (3 months old), suggesting a more direct effect of artemisinin on gephyrin expression than a downstream effect of an ameliorated Aβ-dependent pathology [143]. Since CDK5-dependent phosphorylation at Ser270 may result in higher gephyrin and GABA_A_R-γ2 receptor densities at postsynaptic membrane specializations, this finding also indicates a supporting function of low-dose artemisinin treatment on inhibitory synapse structure. In a more resent study, we reported the modulation of the glycinergic inhibitory system after artesunate treatment in APP/PS1 mice. Analy-zing the glycine receptor (GlyR) α1, α2 and α3 subunit distribution in hAPPswe-expressing cultured hippocampal neurons and brain slices of 12-month-old APP/PS1 mice, we demonstrated that artesunate (10 mg/kg) can rescue the, probably, Aβ-induced loss of extrasynaptic GlyR α3 clusters both in vitro and in vivo, which are thought to play a role in tonic inhibition, an important mechanism in controlling neuronal excitability [144]. Altogether, these few studies indicate that artemisinins can maintain or restore inhibitory synapse protein expression levels in animal models of AD, very likely involving a direct influence on gene expression rather than indirect effects through modulation of other pathogenic mechanisms involved in AD, such as amyloid pathology or reduced cell death. Moreover, fewer data are available concerning the effects of artemisinins on excitatory synapses. Zhou et al. reported that Artemisia annua extract (6.7 mg/mL and 20 mg/mL) elevated the expression levels of synaptophysin, a protein found in presynaptic terminals, and PSD95, a key postsynaptic protein of excitatory synapses, above wild-type levels in the brains of 9-month-old 3xTg AD mice [85]. In our own studies, a significant increase in the PSD95 mRNA level was measured in APP/PS1 mice brains upon treatment with 100 mg/kg artesunate compared to the control APP/PS1 mice [77]. The maintenance of synapses after other artemisinins was also reported; in 9-month-old APPswe/PSEN1dE9 mice, DHA (20 mg/kg/day) upregulated the level of synaptophysin in brain homogenates and promoted neurite outgrowth [117], and in mice injected with Aβ_25–35_ into the lateral ventricle, artemisinin B inhibited synaptophysin loss in the neurons of the hippocampal CA1 region [95].

Taken together, a limited number of studies have so far demonstrated that the reduced expression levels of proteins from both excitatory and inhibitory synapses in different AD models are restored upon treatment with artemisinin compounds. In support of this, in a streptozotocin-induced AD–diabetes rat model [145] and in a mouse model overexpressing human tau (hTau) in the hippocampus, improved synaptic plasticity indicated by increased long-term potentiation (LTP) was reported after artemisinin and DHA treatment, respectively, with improved learning and memory tests. Interestingly, the latter study suggested, as an underlying mechanism for this finding, a DHA-induced modulation of the crosstalk between O-GlcNAcylation and the phosphorylation of tau [146].

### 4.8. Neurogenesis

Adult neurogenesis, comprising the proliferation of progenitor cells derived from neural stem cells upon asymmetric division and their subsequent migration, differentiation into neurons or glia cells and maturation, contributes to the homeostasis of the central nervous system. Cell morphology, expression of transcription factors and a set of marker proteins allow for distinguishing the different stages occurring during the transition from stem cell to mature neuron [147]. The evidence supports a deregulated adult neurogenesis in Alzheimer’s disease associated with learning and memory deficits. It seems to be an early event in Alzheimer’s disease pathogenesis, mediated by intracellular Aβ oligomers [148]. A progressive decline in neurogenesis in AD brains was detected and correlated with disease progression [149], since interventions that promote neurogenesis have been found to alleviate disease symptoms [150,151]. Therefore, it is important to point out a recent study that reported improved neurogenesis in 12-month-old 3xTg AD mice after 3 months of treatment with an extract of Artemisia annua (6.7 mg/mL and 20 mg/mL) containing artemisinin acid, arteether and deoxyartemisinin, correlating with ameliorated cognitive deficits. Specifically, the proliferation and survival of neuronal progenitor cells were promoted. as indicated by significantly increased numbers of Sox2^+^ and Brdu^+^ cells in the hippocampus and cortex of the treated mice [85].

### 4.9. Blood–Brain Barrier

The blood–brain barrier (BBB) is composed of endothelial cells sealed by tight junctions, pericytes and astrocytes, and it is crucial for Aβ homeostasis including clearance dynamics. Structural and functional alterations in the BBB, specifically its endothelial cells, are part of the early pathology of AD brains [152]. The BBB controls the entry of Aβ from plasma into the brain via the receptor for advanced glycation end products (RAGE), whereas the low-density lipoprotein receptor-related protein (LRP1) promotes the removal of brain-derived Aβ. The expressions of both receptors were found to be altered in AD [152,153]. Interestingly, despite suppressed NF-κB-mediated inflammatory signaling and decreased neuritic plaque burden, Shi et al. could not detect significant changes in peripheral blood Aβ_42_ concentration and expression levels of LRP1 and RAGE in brains with relative early pathology in 6-month-old APPswe/PS1dE9 mice after a 30-day treatment with artemisinin (40 mg/kg/day) [72]. In contrast, very recently, Kisler et al. reported that early artesunate (32 mg/kg/day) treatment for 2 months increased blood serum Aβ_42_ and Aβ_40_ levels by approximately two-fold, suggesting the accelerated clearance of Aβ from brain-to-blood in 5-month-old 5XFAD mice that correlated with a reduced Aβ pathology and improved behavioral performance in cognition tests [76]. In this study, it was demonstrated that artesunate specifically increased endothelial PICALM (phosphatidylinositol-binding clathrin assembly protein) levels, a protein that interacts with LRP1 and is involved in internalization of cell surface receptors, as well as in intracellular trafficking of different proteins [154]. It is noteworthy that a 60% reduction in PICALM endothelial levels was measured in human AD brains, correlating inversely with the Aβ load, Braak stage, and clinical dementia, leading to the proposal of a change in the endothelial PICALM level as a significant susceptibility factor for late-onset AD [154].

### 4.10. Memory and Cognition

Qiang et al. reported, in 2018, that artemisinin B parallelly reduced neuroinflammation and significantly improved learning and memory abilities of dementia mice in the Morris water maze (MWM) test, including navigation and space exploration experiments, as well as the step-through test, which analyzes the characteristics of mice that prefer dark places and avoid light [95]. In the same study, the open field test, broadly used to evaluate spontaneous activity and exploratory behavior in mice, showed that the drug had no obvious excitatory or inhibitory effects on the mental state and activity of mice. Shortly thereafter, Li et al. reported results of Morris water maze tests in 10-month-old 3xTg AD mice after artemether therapy. Since untreated 3xTg-AD mice showed significant impairment in learning tasks compared to the wild-type mice, with longer times needed to find the hidden platform, artemether treatment, both at 5 and 20 mg/kg/day doses, significantly shortened the swimming distance of 3xTg-AD mice and decreased the escape latency in comparison to untreated 3xTg AD mice [82]. Thus, artemether treatment rescued spatial learning and reference memory deficits in the 3xTg AD mouse model. These findings were later corroborated by several other studies in different mouse and rat AD models and for various artemisinin compounds. Our literature research identified altogether twelve publications reporting beneficial effects of artemisinins on memory and cognition in AD models that are summarized in Table 1. Most of these studies tested learning and memory abilities using the MWM test, the classic behavioral experiment studying primarily long-term memory, which is affected more in later stages of the disease [155]. In one very recent study, the behavioral performances of 5-month-old 5xFAD mice was evaluated using novel object location, recognition, nesting, and burrowing tests that were improved by artesunate and was correlated with increased expression of a key protein involved in regulating the trans-endothelial transport and clearance of Aβ (PICALM) in brain capillaries [66]. Altogether, these studies provide evidence that the positive modulation of various pathogenic components of AD by artemisinins in rodent models is translated into improvements in memory and cognitive deficits.

## 5. Artemisinins Toxicity

Artemisinin compounds have been in clinical use for more than two decades, and several clinical studies support their high safety and efficacy during the treatment of malaria. The most commonly reported adverse reactions in malaria patients are gastrointestinal nature, including nausea, vomiting, diarrhea, and transient transaminase elevation [156,157]. However, animal and in vitro studies have shown that high doses of artemisinins can be neurotoxic [158,159]. One explanation might be that since human subjects were administered doses of 2–8 mg/kg body weight per day for 3–5 days, most studied animals have been exposed to much higher doses for longer periods of time. The dose causing neurotoxicity or death in 50% of adult mice (Swiss albino) was approximately 300 mg/kg/day for oral artemether and artesunate in comparison to 50 mg/kg/day of intramuscular artemether, both administered for 28 days [158]. In this context, it should be emphasized that APPswePSEN1dE9/Nju mice after oral DHA treatment for 6 months at relatively high doses of 50 and even 300 mg/kg/day exhibited improved behavior in the MWM and open field tests compared to untreated AD mice. Furthermore, in this study, no toxic effects of the DHA treatment were detected on the liver, as evidenced by the unchanged aspartate transferase and alanine aminotransferase levels and on the kidneys, and blood lipid levels were evenly suppressed [74]. In comparison, artesunate at 40 mg/kg administered as a single dose or at 13.4 mg/kg/day given for 3 days at 24 h intervals (i.v.) was found to exert toxic effects on spermatozoa at the morphological and molecular levels in Swiss albino mice, probably involving oxidative mechanisms [160]. Moreover, another study demonstrated that the treatment of zebrafish with artesunate at high doses resulted in acute cardiotoxicity, whereas a low-dose treatment exerted cardioprotective effects [161]. Altogether a biphasic dose-dependent response generated by artemisinins seems to emerge, characterized by beneficial effects at low doses and inhibitory or toxic effects on cellular processes at high doses. However, even this hormetic behavior is strongly context dependent.

Thus, one can conclude, that the bioactivities of artemisinins depend on multiple factors, including dosage, time, drug delivery routes, target cell or tissue types and, probably, from compound chemical characteristics and even species differences [162]. Further, preclinical and clinical studies on pharmacokinetics, long term effect/toxicity and drug–drug interactions are required to understand the reasons for the discrepancies in artemisinin-compounds effects and eventually find the optimal dose in humans with AD that can be administered for long term without toxic effects.

## 6. Conclusions and Future Directions

A relatively large number of well-conducted studies indicate the beneficial effects of artemisinins in preclinical settings of AD, evidencing improvements in pathological hallmarks and pointing to multiple pathogenetic targets within disease development, whose modulation, ultimately, resulted in improved cognitive functions. Different doses of artemisinin and its derivative were tested and found to be effective in the same or different models of AD, which altogether strengthens their therapeutic potential as multitarget drug candidates for a complex disease such as AD. At the same time, the lack of concerted validations of doses and specific efficacies for any artemisinin compound, as well as comparisons of their relative potencies in different animal models, make a direct translation into clinical trials difficult. However, the very context-dependent activity of artemisinins strongly suggests that questions concerning dosing regimens, safety over long-term use, and possible interactions with existing medications, as well as toxicities that could eventually occur linked to treatment in AD patients can be answered appropriately only by well conducted clinical trials.

## Figures and Tables

**Table 1:** Summary of the studies and their main findings from testing different artemisinins in in vivo and in vitro AD models. Doses, route and period of administration, as well as the start of therapy applied in animal models or concentrations used in cell culture experiments, are included. The minimal doses or concentrations to achieve a significant improvement are also shown. Changes induced by the therapy in the given hallmarks of AD are indicated by ↓ (decrease) or ↑ (increase). The arrows in the first row illustrate the changes that occur under AD conditions in comparison to healthy conditions. Key molecular pathways or protein expression alterations are indicated by (+) (increase) or (−) decrease. i.p.—Intra-peritoneally; i.g.—intragastric; p.o.—per oral.

Aβ	pTau	Inflammation	ROS	Autophagy	Cell Death	Synapse Proteins	Neurogenesis	BBB	Memory Cognition	Substance	Dose/Day, Route and Duration of Administration/ Concentration	Lowest Efficient Dose/ Concentration	Molecular Mechanisms		System/Start of Therapy	References
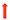	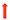	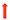	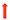	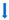	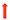	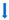	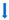	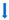	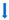	-	-	-	-		-	-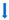		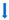								Artemisinin	40 mg/kg, i.p., 30 days	40 mg/kg	NF-κB, IL-6, TNF-α	(−)	APPswe/PS1dE9 mice, 4-month-old	Shi et al., (2013) [72]
													NALP3	(−)		
													BACE1	(−)		
			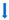		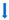					Artemisinin	3.1–100 μM	1.5 μM	ERK1/2 pathway	(+)	PC12 cells + Aβ_25–35_	Zeng et al., (2017) [118]
													Caspase-3 and -7	(−)		
		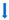				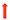			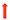	Artemisinin B	20-, 40-, and 80 mg/kg, i.g., 1–2 weeks 1–8 μM	20 mg/kg 1 μM	TLR4, MyD88, NF-κB	(−)	KM mice- intra-ventricular Aβ_25–35_ start:immediately after surgery BV2 cells	Qiang et al., (2018) [95]
													IL-1β, IL-6, TNF-α	(−)		
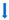	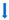		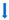		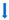				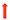	Artemether	5- and 20 mg/kg, i.p., 4 weeks, 3–100 μM	5 mg/kg 10 μM	AMPK/GSK3β/Nrf2	(+)	3xTg mice, 8-month-old PC12 and SH-SY5Y, primary cortical neurons	Li et al., (2019) [82]
													Bcl-2	(+)		
													Bax	(−)		
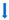	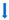	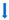	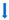		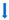				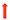	Artemisinin	1-, 5- and 10 mg/kg, i.p., 30 days 3.125–100 μM	1 mg/kg, 6.25 μM	ERK/CREB pathway	(+)	3xTg mice, 11-month-old SH-SY5Y cells	Zhao et al., (2020) [84]
													Cytochrome C	(−)		
													Caspase-3 and -9	(−)		
													Bcl-2/Bax	(+)		
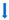				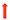					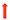	Dihydro-artemisinin	20 mg/kg, p.o., 90 days	20 mg/kg	BACE1	(−)	APPswe/PSEN1dE9, 6-month-old N2a-APP and SH-SY5Y-APP cells	Zhao et al., (2020) [73]
													mTOR/ ULK1	(+)		
					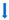	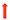				Dihydro artemisinin	20 mg/kg. p.o., 90 days	20 mg/kg	Caspase-3, Bax.	(−)	APPswe/PSEN1dE9, 6-month-old	Zhao et al., (2020) [117]
													Bcl-2	(+)		
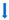						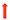				Artemisinin, Artesunate	10- and 100 mg/kg, p.o., 3 months 0.05, 0.125, 0.25 μM	10 mg/kg 0.05 μM	CTFs	(−)	APP_swe_/PS1_L166P_, 9-month-old Hippocampal neurons	Kiss et al., (2021) [77]
						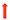				Artemisinin	10- and 100 mg/kg, p.o., 6 weeks	10 mg/kg	Gephyrin phosphorylation	(+)	APP_swe_/PS1_L166P_, 6-week-old	Kiss et al., (2021) [143]
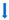		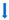	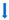							Artemisinin	50 mg/kg, i.p., 4 weeks	50 mg/kg	ROS, TNF-α	(−)	Streptozotocin-induced AD and diabetes in rats	Poorgholam et al., (2021) [103]
													Blood glucose	(−)		
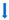	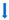								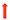	Artemether	n.n.	n.n.	BACE1, mTOR	(−)	Aβ_25–35_-treated rats N2a cells	Li et al., (2021) [83]
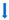		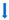	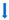	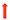	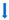				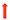	Artesunate	5- and 10 mg/kg, p.p., 6 months 0.01, 0.05, 0.1, 0.2, 0.5, 1.0 μM	5 mg/kg 0.1 μM	TNFα, IL-6, IL-1β	(−)	APPswe/PS1, 2-month-old BV-2-, N2a cells	Qin et al., (2022) [75]
													PINK1.	(+)		
													Parkin	(+)		
		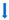	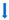		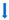				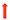	Artemisinin	5 mg/kg, i.p., 4 weeks 0.25, 0.5, 1.0 μM	5 mg/kg 0.25 μM	TLR4/NF-κB	(−)	C57 mice injected with Aβ_1–42_ into the hippocampus, start:4 weeks pre BV2 Cells	Zhao et al., (2022) [96]
													TNF-α, IL-1β, IL-6	(−)		
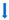	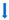								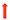	Dihydro-artemisinin	50- and 300 mg/kg, p.o., 4/ 6 months	50 mg/kg	-		APPswePSEN1dE9/Nju, 3-month-old	Xiao et al., (2022) [74]
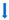		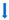						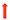	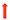	Artesunate	32 mg/kg, i.p., 2 months, 3 μM	EC_50_: 2.1 μM	Brain capillary PICALM	(+)	5XFAD mice, 3-month-old HEK293t luciferase reporter line	Kisler et al., (2023) [76]
						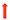				Artesunate	10- and 100 mg/kg, p.o., 1.5/3 months	10 mg/kg	-		APP_swe_/PS1_L166P_, 1.5-/ 9-month- old	Kuhse et al., (2023) [144]
						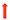			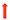	Artemisinin	50 mg/kg, i.p., 4 weeks,	50 mg/kg;	Blood glucose	(−)	Streptozotocin-induced AD and diabetes in rats, 4-month-old	Poorgholam et al., (2023) [145]
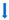	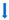	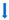	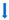		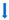	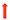	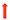		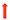	Artemisia annua extract	6.7- and 20 mg/mL, p.o., 3 months 1–10,000 μg/mL	6.7 mg/mL 30 μg/mL	IL-6, TNF-α, IL1β	(−)	3xTg mice, 9-month-old SH-SY5Y, primary neurons PC12 cells	Zhou et al., (2023) [85]
													Hippo/YAP signaling	(+)		
													Bax	(−)		
													Bcl-2	(+)		
